# Proximal Arterial Occlusion in Acute Ischemic Stroke with Low NIHSS Scores Should Not Be Considered as Mild Stroke

**DOI:** 10.1371/journal.pone.0070996

**Published:** 2013-08-16

**Authors:** Joon-Tae Kim, Man-Seok Park, Jane Chang, Ji Sung Lee, Kang-Ho Choi, Ki-Hyun Cho

**Affiliations:** 1 Department of Neurology, Cerebrovascular Center, Chonnam National University Hospital, Gwangju, Korea; 2 Research Institute of Medical Sciences, Chonnam National University Medical School, Gwangju, Korea; 3 Biostatistical Consulting Unit, Soonchunhyang University Medical Center, Seoul, Korea; University of Cambridge, United Kingdom

## Abstract

**Background:**

Untreated acute mild stroke patients have substantial 90-day disability rates and worse outcomes than those who are treated with thrombolysis. There is little information regarding which patients with acute mild stroke will benefit from thrombolysis. We sought to investigate factors that are associated with early neurological deterioration (END) and poor prognosis in patients with acute mild stroke.

**Methods:**

This was a retrospective study of consecutively registered patients with acute mild stroke (NIHSS ≤3) at our tertiary stroke center between October 2008 and December 2011. END was defined as an increase in NIHSS ≥2 points between hospital days 0 and 5. Modified Rankin Scale (mRS) scores of 0–1 at 90 days post-stroke were defined as favorable outcomes.

**Results:**

A total of 378 (mean age, 65.9±13.0 years) patients were included in this study. END occurred in 55 patients (14.6%). IV-thrombolysis was performed in only 9 patients. Symptomatic arterial occlusion on the initial MRA was independently associated with END (OR, 2.206; 95% CI, 1.219–3.994; *p* = 0.009) by multivariate logistic regression. Of the 119 patients with symptomatic arterial occlusion, ICA occlusion was independently associated with END (OR, 8.606; 95% CI, 2.312–32.043; *p* = 0.001).

**Conclusions:**

This study demonstrates that symptomatic arterial occlusion may be an important predictor of END in patients with acute mild stroke. It may therefore be important to consider that acute ischemic stroke with symptomatic arterial occlusion and low NIHSS scores may not represent mild stroke in acute periods.

## Introduction

Ischemic stroke with mild symptoms has typically been associated with good prognosis [1]. However, previous studies have shown that patients with acute mild stroke have substantial 90-day disability rates,[2] and that untreated patients experience worse outcomes than those who are treated with thrombolysis [3], [4]. However, it remains unclear whether thrombolysis may be beneficial for acute mild stroke. There is little information regarding identifying patients with acute mild stroke who will benefit from thrombolysis. Small case series have reported that thrombolysis is effective in acute mild stroke with perfusion defects and arterial occlusion [5], [6]. However, these findings remain inconclusive due to limited small case series.

Early neurological deterioration (END) is associated with poor outcomes, regardless of its definition [7], [8]. Therefore, patients who are at risk of END require more meticulous management. So which patients with acute mild stroke more frequently develop END? A previous report has shown that persistent large vessel occlusion increases the risk of early worsening and poor outcomes [4]. However, this should be interpreted with caution due to the small sample size of this study. Furthermore, it remains unclear which treatments are effective in patients with acute mild stroke who also experience END. Although appropriate management before or after END in patients with acute mild stroke may be important for determining prognosis, a complete understanding of this is lacking.

In this study, we sought to identify imaging characteristics that are useful for predicting END and to evaluate factors that are associated with END and poor prognosis in patients with acute mild stroke.

## Methods

### 1. Patients

This was a retrospective study of prospectively registered patients with acute ischemic stroke at our tertiary stroke center between October 2008 and December 2011. The patients were those who (1) presented and were evaluated within the first 6 hours of symptom onset; (2) underwent emergency stroke magnetic resonance imaging (MRI); and (3) had positive lesions visualized using diffusion-weighted imaging (DWI). We defined mild stroke as occurring in patients who obtained a National Institutes of Health Stroke Scale (NIHSS) score of ≤3.7 Patients obtaining a NIHSS score of 4–5 were considered to be a comparison group of interest [2]. We excluded patients with (1) other etiologies, such as vasculitis, Moyamoya disease or cancer-related stroke; (2) loss of follow-up evaluations; and (3) a previous modified Rankin Scale of >1.

### 2. Ethics Statement

This study was approved by the Institutional Review Board (IRB) of Chonnam National University Hospital. Written informed consent was not obtained from participants because of the retrospective design of this study; therefore, the IRB of the hospital waived the need for written informed consent from participants.

### 3. Imaging studies

According to our stroke imaging protocol, patients underwent emergency MRI at the Emergency Department immediately after hospital admission. The MRI protocol consisted of DWI, fluid-attenuated inversion recovery (FLAIR), gradient echo (GRE) imaging, time-of-flight MR angiography (MRA) and perfusion-weighted imaging (PWI) in sequence. Routine follow-up imaging (DWI/GRE) was performed 3–5 days after symptom onset. In addition, DWI/GRE was also performed if neurologic deterioration was observed.

The images were analyzed by 2 neurologists (J.–T. K. and M.–S. P.) who were blinded to the clinical data. Discrepancies were resolved by consensus. Arterial occlusion was defined as a complete loss of distal flow signal. Moderate to severe arterial stenosis was defined as a >50% narrowing of the lumen and focal signal loss in the presence of a distal flow signal. Arterial occlusion sites that were relevant to ischemia were defined as ‘symptomatic arterial occlusion’ and determined via analysis of the initial MRA, which included the proximal and distal internal carotid artery (ICA), proximal middle cerebral artery (pMCA), distal MCA (dMCA), vertebral arteries (VA), basilar artery (BA) and other arteries (anterior cerebral arteries, posterior cerebral arteries or cerebellar arteries). The proximal MCA was defined as a segment of M1 and the distal MCA was defined as segments beyond M1. The lesion sites visualized via DWI were categorized into anterior, posterior or both circulations. The patterns of initial DWI lesions in anterior circulation were classified as perforating artery infarcts (PAI), pial infarcts (PI), border-zone infarcts (BI), territorial infarcts (TI) and lacunar infarcts (LI), by modification of previous studies [9]. Although all of the patients routinely completed PWI, PWI was not used in the analysis.

### 4. Clinical assessment

Demographic, clinical and laboratory data were collected from the prospectively collected stroke registry. The following stroke risk factors were identified: age, sex, current cigarette smoking status (cigarette smoking within the last 5 years), hypertension, diabetes mellitus, dyslipidemia, and a previous history of stroke or TIA. Baseline data collected from all of the patients included NIHSS scores, admission blood glucose levels, and onset-to-treatment times. Stroke subtypes were stratified according to the Trial of Org 10172 in Acute Stroke Treatment (TOAST) criteria after complete diagnostic profiling [10]. We assessed neurological status at admission and on each hospital day using the NIHSS. Although this study was retrospective, END was carefully assessed by frequent neurological examination.

### 5. Outcome measurements

‘END’ was defined as an increase in NIHSS scores by 2 or more points (or the development of new neurological symptoms, meaning that a subcomponent of the scale that was previously scored a 0 is subsequently scored 1 or more points) and ‘severe END’ was defined as an increase of 4 or more points between hospital days 0 and 5. END was regularly diagnosed and registered by the physician during patient monitoring. We used the combined definitions of END based on previous studies [11], [12]. The patients were divided into 2 groups: those with END and those without END (or severe END). mRS scores of 0–1 at 90 days were defined as favorable outcomes.

### 6. Statistical analysis

The percentage, mean (standard deviation, SD), or median (interquartile range, IQR) are reported depending on variable characteristics. Categorical variables were analyzed using the χ2-test and Fisher's exact test when appropriate. Continuous variables were analyzed using the independent samples *t*-test or the Mann–Whitney *U* test when appropriate (baseline NIHSS scores, onset to visit time, and initial blood glucose levels were analyzed using the Mann-Whitney *U* test after testing for normality). To evaluate the occlusion site as a predictor of END, arterial occlusion sites were adjusted by covariates of END in 119 patients with symptomatic arterial occlusion. In addition, multiple logistic regression analysis was used to evaluate independent factors associated with END and mRS scores >1 at 90 days; model 1 was adjusted by age, sex, initial NIHSS score and symptomatic arterial occlusion; model 2 was adjusted by variables of clinical significance (P<0.2 by univariate analysis and admission blood glucose levels). The associations between END and each occlusion site in 119 patients with symptomatic arterial occlusions were also analyzed. Adjustment was made for the variables of age, female gender, and initial NIHSS scores. Odds ratios (ORs) and 95% confidence intervals (CIs) were calculated. A *p* value of <0.05 was considered to be statistically significant. All of the statistical analyses were performed using SPSS for Windows, version 17 (SPSS Inc., Chicago, IL, USA).

## Results

### 1. General characteristics

A total of 399 patients with acute mild stroke were screened within 6 hours of symptom onset for the study period. Of these patients, 21 were excluded: 8 were excluded due to lost follow-up, 5 were excluded due to incomplete work-up, 4 were excluded due to a previous mRS score of >1, 3 were excluded due to other etiologies, including venous infarcts, and 1 was excluded due to ischemic stroke combined with hemorrhagic stroke. Ultimately, 378 patients (227 men and 151 women, mean age, 65.9±13.0 years) who obtained NIHSS scores >4 were analyzed. Among them, 311(82.3%) patients obtained mRS scores of 0–2 at 3 months and 250 (66.1%) obtained mRS scores of 0–1 at 3 months. END and severe END occurred in 55 (14.6%) patients and 28 (7.4%) patients, respectively. IV-thrombolysis was performed in only 9 of these patients.

In the same period, 134 patients who were screened within 6 hours of symptoms onset obtained NIHSS scores of 4 or 5. The frequency of END was not significantly different between patients who obtained an initial NIHSS score of 0 to 3 and those obtaining an initial NIHSS score of 4–5 (14.6% vs. 20.1%, p = 0.133). However, when patients who obtained an NIHSS score of 4 or 5 were divided into those who received thrombolysis and those who did not, patients who had not received thrombolysis (N = 72) were less frequently associated with a mRS score of 0–2 and mRS score of 0–1 at 3 months than those with an NIHSS score of 0 to 3 (33.3% vs. 66.1%, p<0.001) (Table S1).

### 2. The characteristics of patients with END and factors associated with END

Table 1 presents the general characteristics and angiographic features of patients with END and those without END. END more frequently occurred in females. Large artery atherosclerosis in the TOAST classification and symptomatic arterial occlusion developed more frequently in patients with END. In addition, favorable outcome at 3 months was less frequently observed in patients with END than in those without. The rates of thrombolytic treatment were not significantly different between patients with END and those without END. Factors that were independently associated with END were assessed by a multivariate logistic regression model, adjusted for variables with clinical significance. Table 2 presents the results of this analysis. Symptomatic arterial occlusion was an independent factor associated with severe END and END (Model 1: OR, 5.945 [2.493–14.175] and 2.206 [1.219–3.994], Model 2: OR, 6.124 [2.444–15.344] and 2.442 [1.283–4.649], respectively).

**Table 1:** General characteristics of subjects.

	Severe END (N = 28)	No severe END (N = 350)	*p*	END (N = 55)	No END (N = 323)	*p*
Age (mean ± SD)	68.17±14.00	65.66±12.89	0.325	68.14±13.45	65.46±12.86	0.157
Male (n, %)	11 (39.3)	216 (61.7)	0.026	26 (47.3)	201 (62.2)	0.052
Risk factors (n, %)						
Hypertension	19 (67.9)	204 (58.3)	0.425	35 (36.6)	188 (58.2)	0.464
Diabetes	7 (25.0)	103 (29.4)	0.829	15 (27.3)	95 (29.4)	0.873
Dyslipidemia	9 (32.1)	112 (32.0)	>0.999	21 (38.2)	100 (31.0)	0.348
Atrial fibrillation	8 (28.6)	70 (20.0)	0.330	10 (18.2)	68 (21.1)	0.721
Smoking	4 (14.3)	95 (27.1)	0.181	8 (14.5)	91 (28.2)	0.045
Previous stroke or TIA	4 (14.3)	55 (15.7)	>0.999	7 (12.7)	52 (16.1)	0.688
TOAST classification (n, %)			0.347			0.010
LAA	15 (53.6)	147 (42.0)		35 (63.6)	127 (39.3)	
CE	7 (25.0)	77 (22.0)		10 (18.2)	74 (22.9)	
SVO	0	46 (13.1)		1 (1.8)	45 (13.9)	
UD	6 (21.4)	80 (22.9)		9 (16.4)	77 (23.8)	
Baseline NIHSS (med, IQR)	2.0 (1.75)	2.0 (1.0)	0.023	2.0 (2.0)	2.0 (1.0)	0.345
Onset to visit time (med, IQR)	157.0 (148.75)	138.5 (142.0)	0.946	160.0 (115.0)	134.0 (145.0)	0.580
Blood glucose level (med, IQR)	127.0 (44.25)	124.0 (48.5)	0.303	120.0 (47.0)	125.0 (49.0)	0.919
Lesion location (n, %)			0.296			0.924
Anterior circulation	22 (78.6)	234 (66.9)		39 (70.9)	217 (67.2)	
Posterior circulation	5 (17.9	106 (30.3)		13 (23.6)	98 (30.3	
Both circulations	1 (3.6	10 (2.9)		3 (5.5)	8 (2.5)	
Lesion patterns (N = 256)*						
PAI (N = 11)	2 (5.1)	9 (4.1)	0.677	2 (9.1)	9 (3.8)	0.242
PI (N = 95)	9 (40.9)	86 (36.8)	0.818	13 (33.3)	82 (37.8)	0.719
BI (N = 39)	5 (22.7)	34 (14.5)	0.348	8 (20.5)	31 (14.3)	0.335
TI (N = 46)	6 (27.3	40 (17.1)	0.247	9 (23.1)	37 (17.1)	0.369
LI (N = 99)	3 (13.6)	96 (41.0)	0.011	11 (28.2)	88 (40.6)	0.158
Combined (N = 29)	3 (13.6)	26 (11.1)	0.724	4 (10.3)	25 (11.5)	>0.999
Angiography (n, %)						
Symptomatic artery			<0.001			0.034
No steno-occlusion	6 (21.4)	173 (49.4)		22 (40.0)	157 (48.6)	
Stenosis	2 (7.1)	78 (22.3)		7 (12.7)	73 (22.6)	
Occlusion	20 (71.4)	99 (28.3)		26 (47.3)	93 (28.8)	
Irrelevant artery			0.547			0.683
Stenosis	4 (14.3)	42 (12.0)		10 (18.2)	36 (11.1)	
Occlusion	2 (7.1)	18 (5.1)		2 (3.6)	18 (5.6)	
Thrombolysis (n, %)	1 (3.6)	8 (2.3)	0.504	1 (1.8)	8 (2.5)	>0.999
mRS score 0–2 at 90 days	6 (21.4)	305 (87.1)	<0.001	21 (38.2)	290 (89.8)	<0.001
mRS score 0–1 at 90 days	5 (17.9)	245 (70.0)	<0.001	14 (25.5)	236 (73.1)	<0.001

Baseline NIHSS scores, onset to visit time, and initial blood glucose levels were analyzed using the Mann-Whitney *U* test after tests of normality.

END, early neurological deterioration; TOAST, Trial of Org 10172 in Acute Stroke Treatment; LAA, large artery atherosclerosis; CE, cardioembolism; SVO, small vessel occlusion; UD, undetermined; NIHSS, National Institutes of Health Stroke Scale; PAI, perforating artery infarcts; PI, pial infarcts; BI, border zone infarcts; TI, territorial infarcts; LI, lacunar infarcts; IVT, intra-venous thrombolysis; IAR, intra-arterial revascularization.

* Lesion patterns were analyzed only in patients with lesions in anterior circulation.

**Table 2:** Independent predictors of various types of END by multivariate logistic regression analysis.

	Severe END				END			
	Model 1 (OR, 95% CI)	P	Model 2 (OR, 95% CI)	P	Model 1 (OR, 95% CI)	P	Model 2 (OR, 95% CI)	p
Arterial occlusion	5.945 (2.493–14.175)	<0.001	6.124 (2.444–15.344)	<0.001	2.206 (1.219–3.994)	0.009	2.442 (1.283–4.649)	0.007
Female	2.214 (0.968–5.066)	0.060	1.966 (0.794–4.870)	0.144	1.684 (0.932–3.043)	0.084	1.377 (0.717–2.644)	0.336
Baseline NIHSS	1.365 (0.892–2.090)	0.152	1.315 (0.850–2.036)	0.219	1.038 (0.775–1.390)	0.801	1.016 (0.751–1.375)	0.915
Age	1.010 (0.977–1.044)	0.552	1.012 (0.978–1.047)	0.486	1.014 (0.990–1.039)	0.257	1.016 (0.991–1.041)	0.215
Smoking	NA		0.634 (0.185–2.170)	0.468	NA		0.499 (0.205–1.211)	0.124
Blood glucose	NA		1.003 (0.997–1.010)	0.998	NA		1.000 (0.995–1.006)	0.957
TOAST	NA			0.525	NA			0.003
LAA			1.586 (0.547–4.601)	0.396			2.710 (1.199–6.125)	0.017
CE			0.734 (0.221–2.433)	0.613			0.874 (0.326–2.340)	0.788
SVO			0.000	0.998			0.291 (0.034–2.464)	0.257
UD			Reference				Reference	

Model 1; adjusted by age, sex, and initial NIHSS score.

Model 2 for severe END was adjusted by variables with Model 1, admission blood glucose, smoking and TOAST.

Model 2 for END was adjusted by variables with Model 1, admission blood glucose, smoking and TOAST.

END, early neurological deterioration; TOAST, Trials of Org 10172 in Acute Stroke Treatment; LAA, large artery atherosclerosis; CE, cardioembolism; SVO, small vessel occlusion; UD, undetermined; NA, not applicable.

Of the 119 patients with symptomatic arterial occlusion (Table 3), 20 (17.9%) developed severe END and 26 (24.5%) developed END. ICA occlusion (n = 17) was independently associated with severe and non-severe END (severe END: OR, 6.508; 95% CI, 1.610–26.306; *p* = 0.009, and END: OR, 8.606; 95% CI, 2.312–32.043; *p* = 0.001) by multivariate logistic regression. In 74 patients with MCA and ICA occlusions, the lesion pattern of BI was highly related to severe END but this relationship was not statistically significant (35.7% vs. 16.7%, p = 0.142). The analysis of the patients who obtained NIHSS scores of 0–5 revealed that the BI pattern occurred at high frequency in severe END with borderline statistical significance (38.1% vs. 19.2%, p = 0.083). Other lesion patterns of MCA/ICA occlusion were not related to END or severe END in patients with acute mild stroke.

**Table 3:** Associations between END types and various occlusion sites in 119 patients with symptomatic arterial occlusions.

Occluded arteries (END, severe END)*	Adjusted OR for END (95% CI)	P	Adjusted OR for severe END (95% CI)	P
**ICA occlusion (8/17, 6/17)**	8.606 (2.312–32.043)	0.001	6.508 (1.610–26.306)	0.009
No ICAO	Reference	0.016	Reference	0.091
Distal (1/1, 1/1)	–	–	–	–
Proximal (7/16, 5/16)	7.255 (1.878–28.035)	0.004	5.066 (1.187–21.621)	0.028
**MCA occlusion (12/57, 9/57)**	0.878 (0.361–2.139)	0.857	0.855 (0.319–2.292)	0.755
No MCAO	Reference	0.007	Reference	0.056
Distal (3/38, 0/38)	0.274 (0.072–1.050)	0.059	–	
Proximal (9/19, 9/19)	3.032 (1.010–9.186)	0.050	4.074 (1.294–12.831)	0.016
**VBA occlusion (4/25, 4/25)**	0.617 (0.189–2.010)	0.423	0.936 (0.278–3.147)	0.915
No VBAO	Reference	0.655	Reference	0.866
Vertebral (2/16, 2/16)	0.481 (0.100–2.303)	0.360	0.728 (0.148–3.579)	0.696
Basilar (2/9, 2/9)	0.867 (0.164–4.575)	0.867	1.321 (0.243–7.167)	0.747
**Other occlusion (2/20, 1/20)**	0.285 (0.060–1.361)	0.116	0.172 (0.021–1.419)	0.102

* Numbers in parenthesis: numbers of END/numbers of patients with symptomatic arterial occlusion, numbers of severe END/numbers of patients with symptomatic arterial occlusion, respectively.

Adjusted by age, female, and initial NIHSS scores.

**Figure 1:**
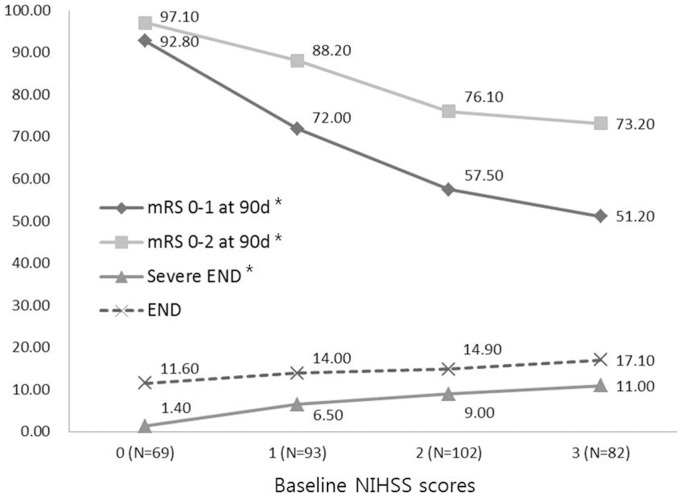
The distribution of various types of END and mRS scores of 0–1 and 0–2 at 90 days in terms of baseline NIHSS scores (^*^ *p* for trends <0.05).

**Figure 2:**
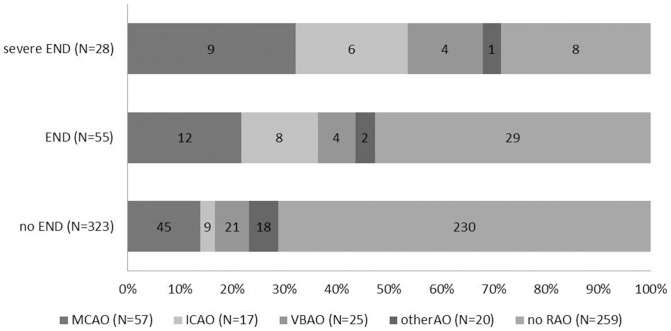
The distribution of occlusion sites according to END type. **MCA and ICA occlusions contributed most frequently to severe END.** (Abbreviation: MCAO, middle cerebral artery occlusion; ICAO, internal carotid artery occlusion; VBAO, vertebrobasilar artery occlusion; otherAO, other arterial occlusion; no RAO, no relevant arterial occlusion).

Baseline NIHSS scores were related to outcomes at 3 months, but not with END (Figure 1). In addition, severe END was more strongly related to symptomatic arterial occlusion (71.4%), especially MCA and ICA occlusion, than END (Figure 2).

### 3. Factors associated with mRS scores >1 at 3 months in patients with mild stroke

Table S2 presents the general characteristics of patients with favorable outcomes at 3 months and those without such outcomes. The rates of thrombolytic treatment were not significantly different between patients with good outcomes and those with poor outcomes. Thrombolysis in patients with NIHSS scores of 0–3 did not affect outcomes and was not associated with END. However, in patients with baseline NIHSS scores of ≥4, those who underwent thrombolysis had more favorable outcomes at 3 months than those who did not (Table S1). Factors that were independently associated with mRS score >1 at 3 months were assessed by a multivariate logistic regression model adjusted for variables with clinical significance (Table S3). Symptomatic arterial occlusion was an independent predictor of poor outcomes at 3 months. Unlike END, the initial NIHSS score was the most important factor associated with mRS scores of >1 at 90 days.

## Discussion

This study demonstrated that symptomatic arterial occlusion on initial MRA could be an important predictor of END and poor outcomes at 3 months in patients with acute mild stroke. In addition, among arterial occlusion sites, ICA occlusion was independently associated with END in acute mild stroke. The results of this study may be useful in informing clinical decision making. If physicians are hesitant to treat a patient with thrombolysis, they may be able to conclude the best course of action based on imaging results. Our study has clinical implications for investigating imaging findings, especially the site of arterial occlusion, as a factor associated with END in patients with acute mild stroke. In addition, it is noteworthy that our study analyzed a larger patient sample than other previous studies.

Patients with mild stroke were analyzed in this study. The definition of mild stroke is not consistent in the literature [2], [4], [13], [14]. We defined mild stroke as a baseline NIHSS score of 0 to 3. Fischer et al [15] found that patients fulfilling this definition had the best medium-term outcomes. Our results support the previously reported result that low baseline NIHSS scores (≤3) are related to more favorable outcomes. However, END was not correlated with baseline NIHSS scores in acute mild stroke. Although admission NIHSS score was a significant predictor of END in other studies, [16] it was not significant among patients with low baseline NIHSS scores in the present study. This may be due to differences in inclusion criteria. As in the present study, a previous report demonstrated that groups with low NIHSS scores experienced lower frequencies of worsening than those with high NIHSS scores [17].

We defined different types of END in this study because there is no standard definition of END in the literature. Because the time of the first assessment after initial stroke onset may influence the frequency of END, this study only included patients with acute ischemic stroke within 6 hours of symptom onset. Previous studies have used either a 2-point or more increase or a 4-point or more increase in NIHSS scores [11], [12]. Although these alternate definitions were used in the present study, we found significant associations between imaging findings and the 2 types of END. Here, we found that imaging findings, especially angiographic characteristics, could predict END in acute mild stroke; however, lesion patterns of anterior circulation were not associated with END, which differs from previously reported findings [18]. However, based on patients with only MCA and ICA occlusion, there was a tendency for BI to frequently develop into severe END. These characteristics were more apparent when increasing the range of patients to include those with NIHSS scores of 0–5. Although there were no statistically significant differences due to small sample sizes, the association between lesion patterns and severe END in patients with MCA and ICA occlusion should be further addressed in future studies. In addition, the frequency of severe END was greater in ICA occlusion and pMCA occlusion (15 of 28 patients with severe END, 53.6%) than in other arterial occlusions. Therefore, patients with ICA occlusion and pMCA occlusion may have more clinically important implications than those with other arterial occlusions. Rajajee et al [4] reported that END is more frequent in proximal artery occlusion, which is supported by our results. The presence of ICA and pMCA occlusion may be important for targeting END prevention in patients with acute mild stroke. Therefore, restoration of cerebral perfusion pressure, and/or combined antiplatelet therapy in acute time periods for the prevention of severe END should be considered [19]–[21]. Therefore, further studies are necessary to confirm whether any strategies may be helpful in treating such patients.

Our study was limited by small sample sizes and could not demonstrate the clinical implications of thrombolysis in acute mild stroke. The efficacy of thrombolysis in acute mild stroke has yet to be determined [22]. In the present study, the patients were neither randomized nor blinded to thrombolysis and the number of thrombolysed patients with a low NIHSS score (0 to 3) was very small. We therefore could not confirm the efficacy of thrombolysis in patients with acute mild stroke. In our country, alteplase is not covered by health insurance in patients with acute mild stroke and an NIHSS score <4. Additionally, physicians in our country usually do not administer IV alteplase in patients with mild stroke and such low NIHSS scores. These considerations warrant further study and confirmatory results are greatly needed in our country. Whether patients with acute mild stroke would benefit from thrombolysis remains controversial, [23] especially those with ICA and pMCA occlusion. Further prospective studies with larger sample sizes are needed to confirm the effectiveness of thrombolysis in patients with acute mild stroke and proximal arterial occlusion.

A previous study found that higher blood glucose levels were associated with END after IV-thrombolysis [24]. The authors of this work postulated that the mechanisms that underlie hyperglycemia induced neurological deterioration, which may be due to endothelial damage, deteriorating tissue acidosis, and worsening blood-brain barrier breakdown [25]–[27]. In contrast to a previously reported study, our study focused on imaging findings as predictors of END in acute mild stroke patients. In the present study, only 46 patients (12.2%) presented with high blood glucose levels at admission (over 200 mg/dL); no significant association was found between admission blood glucose levels and END.

Other limitations were present in the current study. First, this is a retrospective study. Although we consecutively evaluated patients with acute mild stroke according to our stroke protocol, further studies with a prospective design are needed to confirm our results. In particular, we could not confirm the efficacy of thrombolysis in patients with acute mild stroke because the patients were neither randomly selected nor blinded to thrombolysis. However, because it is difficult to perform prospective randomized clinical studies on the efficacy of thrombolysis in acute mild stroke, our results may be of value. Second, the definition of acute mild stroke represents another limitation. Low NIHSS scores do not always represent mild ischemic symptoms [28]. Maas et al [29] have shown that NIHSS scores are poorly predictive of proximal occlusion in acute ischemic stroke. Further studies of NIHSS scores in acute ischemic stroke are warranted.

In conclusion, the results of this study demonstrate that symptomatic arterial occlusion could be a predictor of END in patients with acute mild stroke. Our results suggest that acute mild stroke with proximal arterial occlusion should not initially be considered as mild stroke. Further studies with a prospective design are needed to investigate therapeutic strategies for acute mild stroke with proximal arterial occlusion.

## Supporting Information

Table S1 Comparisons of patients with initial NIHSS ≤3 and 4 to 5.
(DOCX)

Table S2 General characteristics of subjects with mRS >1 at 90 days.
(DOCX)

Table S3 Independent predictors of mRS 0–1 at 90 days by multivariate logistic regression analysis.
(DOCX)
