# Supplementary material for: Proximal Arterial Occlusion in Acute Ischemic Stroke with Low NIHSS Scores Should Not Be Considered as Mild Stroke
Source: PLoS One. 2013 Aug 16;8(8):e70996. doi: 10.1371/journal.pone.0070996 (PMC3745393; doi:10.1371/journal.pone.0070996)
Supplement: Table S1 — Comparisons of patients with initial NIHSS ≤3 and 4 to 5. (DOCX) [file pone.0070996.s001.docx]

Table S1. Comparisons of patients with initial NIHSS ≤3 and 4 to 5

|  | NIHSS 0-3 (N=378) | NIHSS 4-5 (N=134) | p | NIHSS 4-5 | | P^1^ | P^2^ |
| --- | --- | --- | --- | --- | --- | --- | --- |
|  |  |  |  | Thrombolysis (N=62) | No thrombolysis (N=72) |  |  |
| END | 55 (14.6) | 27 (20.1) | 0.133 | 12 (19.4) | 15 (20.8) | 0.341 | 0.213 |
| Severe END | 28 (7.4) | 17 (12.7) | 0.075 | 11 (17.7) | 8 (8.3) | 0.014 | 0.808 |
| mRS 0-2 at 90 days | 311 (82.3) | 88 (65.7) | <0.001 | 47 (75.8) | 41 (56.9) | 0.222 | <0.001 |
| mRS 0-1 at 90 days | 250 (66.1) | 59 (44.0) | <0.001 | 35 (56.5) | 24 (33.3) | 0.152 | <0.001 |

P^1^: comparison of NIHSS 0-3 versus NIHSS 4-5 with thrombolysis

P^2^: comparison of NIHSS 0-3 versus NIHSS 4-5 with no thrombolysis

END, early neurological deterioration; mRS, modified Rankin Scale.
