# Supplementary material for: Proximal Arterial Occlusion in Acute Ischemic Stroke with Low NIHSS Scores Should Not Be Considered as Mild Stroke
Source: PLoS One. 2013 Aug 16;8(8):e70996. doi: 10.1371/journal.pone.0070996 (PMC3745393; doi:10.1371/journal.pone.0070996)
Supplement: Table S2 — General characteristics of subjects with mRS >1 at 90 days. (DOCX) [file pone.0070996.s002.docx]

Table S2. General characteristics of subjects with mRS > 1 at 90 days.

|  | mRS >1 (N=128) | mRS 0-1 (N=250) | *p* |
| --- | --- | --- | --- |
| Age (mean±SD) | 68.7±8.9 | 64.3±13.2 | 0.002 |
| Male (n, %) | 62 (48.4) | 165 (66.0) | 0.001 |
| Risk factors (n, %) |  |  |  |
| Hypertension | 83 (64.8) | 140 (56.0) | 0.122 |
| Diabetes | 49 (38.3) | 61 (24.4) | 0.006 |
| Dyslipidemia | 53 (41.4) | 68 (27.2) | 0.007 |
| Atrial fibrillation | 23 (18.0) | 55 (22.0) | 0.421 |
| Smoking | 24 (18.8) | 75 (30.0) | 0.019 |
| Previous stroke or TIA | 18 (14.1) | 41 (16.4) | 0.654 |
| TOAST classification (n, %) |  |  | 0.529 |
| LAA | 64 (50.0) | 98 (39.2) |  |
| CE | 23 (18.0) | 61 (24.4) |  |
| SVO | 10 (7.8) | 36 (14.4) |  |
| UD | 31 (24.2) | 55 (22.0) |  |
| Baseline NIHSS (med, IQR) | 2.0 (1.0) | 1.0 (2.0) | <0.001 |
| Onset to visit time (mean±SD) | 164.2±98.0 | 154.0±90.6 | 0.419 |
| Admission blood glucose (mean±SD) | 145.2±50.3 | 137.8±55.9 | 0.015 |
| Lesion location (n, %) |  |  | 0.372 |
| Posterior circulation | 43 (33.6) | 68 (27.2) |  |
| Anterior circulation | 80 (62.5) | 176 (70.4) |  |
| Both | 5 (3.9) | 6 (2.4) |  |
| Arterial occlusion (n, %) | 51 (39.8) | 68 (27.2) | 0.014 |
| ICA occlusion | 8 (6.3) | 9 (3.6) | 0.295 |
| MCA occlusion | 20 (15.6) | 37 (14.8) | 0.880 |
| VBA occlusion | 11 (8.6) | 14 (5.6) | 0.280 |
| Other arterial occlusion | 12 (9.4) | 8 (3.2) | 0.015 |
| Thrombolysis (n, %) | 4 (3.1) | 5 (2.0) | 0.494 |

Baseline NIHSS scores, onset to visit time, and initial blood glucose were analyzed using the Mann-Whitney *U* test after test of normality.

END, early neurological deterioration; TOAST, Trial of Org 10172 in Acute Stroke Treatment; LAA, large artery atherosclerosis; CE, cardioembolism; SVO, small vessel occlusion; UD, undetermined; NIHSS, National Institutes of Health Stroke Scale.
