# Supplementary material for: Proximal Arterial Occlusion in Acute Ischemic Stroke with Low NIHSS Scores Should Not Be Considered as Mild Stroke
Source: PLoS One. 2013 Aug 16;8(8):e70996. doi: 10.1371/journal.pone.0070996 (PMC3745393; doi:10.1371/journal.pone.0070996)
Supplement: Table S3 — Independent predictors of mRS 0–1 at 90 days by multivariate logistic regression analysis. (DOCX) [file pone.0070996.s003.docx]

Table S3. Independent predictors of mRS 0-1 at 90 days by multivariate logistic regression analysis

|  | Model 1 for mRS >1 (OR, 95% CI) | P | Model 2 for mRS >1 (OR, 95% CI) | P |
| --- | --- | --- | --- | --- |
| Age | 1.022 (1.001-1.042) | 0.036 | 1.022 (1.002-1.043) | 0.034 |
| Female | 1.694 (1.010-2.842) | 0.046 | 1.710 (1.011-2.894) | 0.046 |
| Arterial occlusion | 1.785 (1.085-2.938) | 0.023 | 2.032 (1.179-3.500) | 0.011 |
| Baseline NIHSS | 1.828 (1.422-2.349) | 0.000 | 1.854 (1.436-2.394) | 0.000 |
| Diabetes | 1.990 (1.099-3.603) | 0.023 | 1.863 (1.017-3.413) | 0.044 |
| Smoking | 0.858 (0.463-1.588) | 0.625 | 0.819 (0.437-1.534) | 0.532 |
| Dyslipidemia | 2.201 (1.341-3.614) | 0.002 | 1.989 (1.197-3.304) | 0.008 |
| Blood glucose | 0.999 (0.994-1.004) | 0.700 | 0.999 (0.994-1.004) | 0.668 |
| Hypertension | NA |  | 1.241 (0.751-2.050) | 0.399 |
| TOAST | NA |  |  | 0.074 |
| LAA |  |  | 1.066 (0.580-1.957) | 0.837 |
| CE |  |  | 0.465 (0.221-0.978) | 0.044 |
| SVO |  |  | 0.659 (0.266-1.637) | 0.369 |
| UD |  |  | Reference |  |

Factors associated with mRS>1 at 90 days: Model 1; adjusted by age, sex, initial NIHSS, diabetes, dyslipidemia, smoking, admission blood glucose and arterial occlusion. Model 2; adjusted by age, sex, initial NIHSS, hypertension, diabetes, smoking, dyslipidemia, admission blood glucose, TOAST classifications and arterial occlusion.

OTV, onset to visit time; TOAST, Trial of Org 10172 in Acute Stroke Treatment; LAA, large artery atherosclerosis; CE, cardioembolism; SVO, small vessel occlusion; UD, undetermined.
